# The association between child abuse and poor academic performance in public primary schools in urban Sudan

**DOI:** 10.1192/j.eurpsy.2023.1840

**Published:** 2023-07-19

**Authors:** B. A. A. Mohammed

**Affiliations:** LIAISON PSYCHIATRY, the mater misericordiae university hospital, Dublin, Ireland

## Abstract

**Introduction:**

Child abuse, sometimes referred to as child abuse and neglect, includes all forms of physical and emotional ill-treatment, sexual abuse, neglect, and exploitation that results in actual or potential harm to the child’s health, development or dignity. Child abuse with all its forms is under reported globally in developed and developing countries. The extent of the problem is difficult to ascertain since most of the victims remain silent thus, assessing its prevalence and effects on children is crucial.

**Objectives:**

To assess the association between child abuse with poor school performance considering related variables including family instability, home environment, and school environment.

**Methods:**

This study included 504 participants: 168 cases and 336 controls. Controls were matched for age and gender. Both groups were tested for marital or family instability, home environment, school environment, peer and teacher maltreatment using bi-variate analysis. Test of significance of the result was estimated by confidence interval. Quantification of each factor effect and the interrelationships between all factors that affect academic performance was calculated using multivariate analysis by multiple logistic regressions.

**Results:**

After controlling for confounding factors using a multivariate analysis model, the following was found: a significant increase in the risk of poor academic performance in children who were subjected to maternal physical neglect compared with children who were not (OR= 3.106: CI 95%=1.875-5.147), and in children subjected to maternal emotional neglect compared with children who were not (OR= 1.968: CI 95% = 1.200- 3.226). No association was found between maternal emotional/verbal or physical abuse and poor academic performance.

There is a significant increase in the risk of poor academic performance in children subjected to paternal emotional verbal abuse compared with children who were not (OR= 4.534: CI 95% = 1.833 - 11.214), but the result illustrated no association between paternal physical abuse and poor academic performance.

**Conclusions:**

The risk of poor academic performance is significantly higher in children exposed to frequent parental conflicts or physical abuse from teachers compared with children who were not. There is a significant increase in the risk of poor academic performance in children who experienced maternal physical and emotional neglect and paternal emotional/ verbal abuse. Higher levels of parental education, feeling safe at home, and feeling happy may be protective factors.

**Disclosure of Interest:**

None Declared

